# Disseminated *Bartonella henselae* Infection Visualized by [^18^F]FDG-PET/CT and MRI

**DOI:** 10.3390/diagnostics9010025

**Published:** 2019-03-01

**Authors:** Marie Norredam, Andreas Knudsen, Carsten Thomsen, Lothar Wiese

**Affiliations:** 1 Department of Infectious Diseases, University Hospital Hvidovre, 2800 Copenhagen, Denmark; 2 Department of Internal Medicine, Zealand University Hospital, 4000 Roskilde, Denmark; jeakn@regionsjaelland.dk (A.K.); low@regionsjaelland.dk (L.W.); 3 Department of Radiology, Zealand University Hospital, 4000 Roskilde, Denmark; cert@regionsjaelland.dk

**Keywords:** disseminated *Bartonella henselae* infection, [^18^F]FDG-PET/CT, MRI

## Abstract

We describe the clinical course of a 24-year old male with Crohn’s disease in immunosuppressive therapy admitted with a 6-week history of fever, weight loss, night sweat, and general malaise. The patient received extensive workup for a fever of unknown origin and received empiric antibiotics. Workup with Fluorine-18 fluoro-2-deoxy-d-glucose ([^18^F]FDG) positron-emission tomography (PET/CT), and magnetic resonance imaging (MRI) with intravenous contrast showed multifocal ostitis of the columna and os sacrum, as well as abscesses in m. iliopsoas and m. iliacus and affection of the retroperitoneum, liver, and spleen. Initially, malignancy was suspected, but a subsequent liver biopsy showed necrotizing granulomatous inflammation and a later polymerase chain reaction (PCR) showed *Bartonella henselae*. The patient had relevant exposure from housecats. He was treated with Doxycycline and Rifampicin for 12 weeks resulting in complete recovery. This case is, to our knowledge, a rare example of disseminated infection with *Bartonella henselae* visualized on both [^18^F]FDG-PET/CT and MRI.

**Figure 1:**
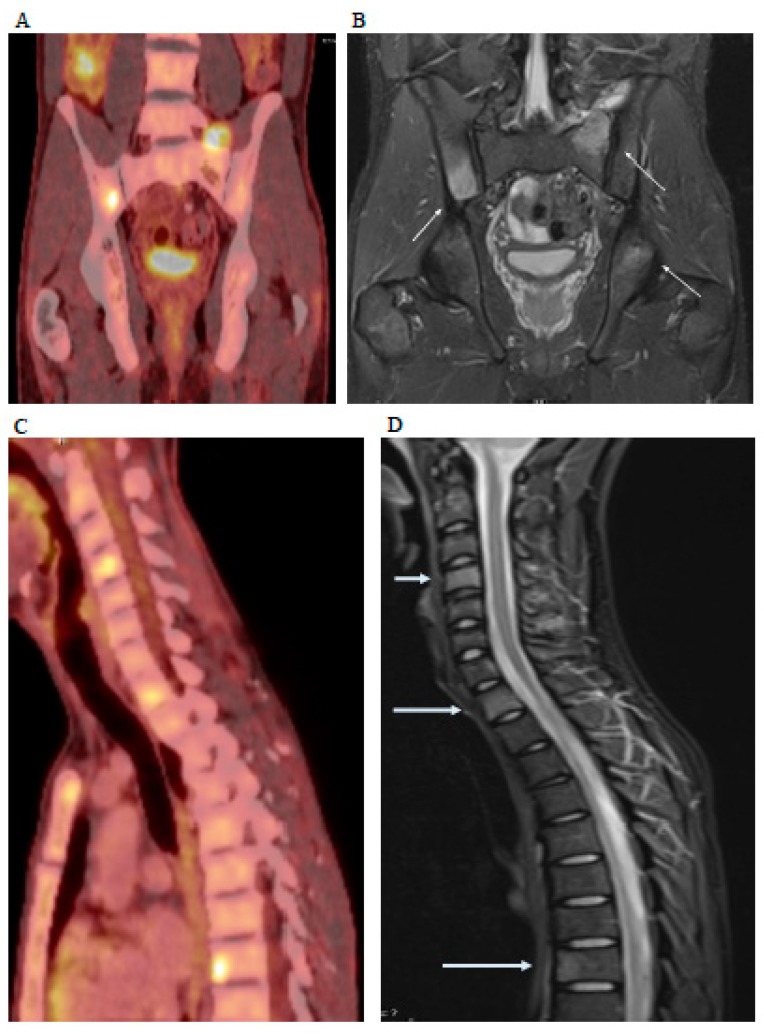
A 24-year old male bricklayer was admitted to a local hospital with a 4-week long history of fever, diarrhea, weight loss, night sweats, and general malaise. The patient had a two-year history of stable Crohn’s disease treated with weekly injections of a TNF-alpha inhibitor. One month prior to admission he had received an additional course of Azathioprine, which was terminated due to side effects. The patient had two domestic cats and a dog. Upon admission, the patient was febrile (39.5 °C) with a blood pressure of 112/60 mmHg. Laboratory test showed leucocytes of 3.0 × 10^9^/L and C-reactive protein of 87 mg/L. The chest X-ray was normal and urine dip stick and urine culture were negative. All blood cultures were negative. He was treated empirically with Piperacillin/Tazobactam and Metronidazole. Consequently, echocardiography (Echo) showed no abnormal findings. Computed tomography (CT) of the thorax and abdomen showed pericardial fluid and moderate ascites compatible with Crohn’s disease. In addition, a solitary infarct-like lesion was seen in the spleen and the liver was enlarged with several poorly defined hypodense lesions up to 1.5 cm. Next, 2-deoxy-2-[^18^F]fluoroglucose ([^18^F]FDG) positron-emission tomography (PET) was performed showing multiple PET positive foci in the liver, spleen, retroperitoneum, and ileocecum (**A**), as well as ostitis in the thoracic (**C**) and lumbar column, sternum, os sacrum, clavicle, and costae. Additionally, a magnetic resonance imaging (MRI) with intravenous contrast confirmed the suspicion of spondylitis in the thoracic column (**D**) revealing additional abscesses in m. iliopsoas and m. iliacus (**B**). After five weeks the patient was referred to the department of infectious diseases, on referral, the patient was marked by chronic disease and febrile (38.0 °C). A liver biopsy showed necrotizing granulomatous inflammation, no mycobacteria, fungi, or bacteria and negative immunohistochemistry for spirochetes as well as CD30 without Hodgkin cells. Subsequent PCR from the biopsy showed *Bartonella henselae* but no *Borrelia burgdoferi* or *Treponema pallidum*. A subsequent serology test for *Bartonella henselae* was highly positive. The treatment was changed to Doxycycline and Rifampicin and the patient received a total course of 12 weeks treatment of which the first 6 weeks were intravenous. The clinical symptoms resolved after 6 weeks of i.v. antibiotic and full recovery was achieved after a total of 12 weeks’ AB. This case shows the importance of considering unusual infection when fever and systemic symptoms do not resolve with empirical antibiotics. Second, it underscores the ability of immunosuppressive treatment to pave the way for atypical infections [1,2] which can be difficult to diagnose by routine sampling, not covered by empiric antibiotics and disseminate as clearly shown by both [^18^F]FDG-PET/CT and MRI. Third, as previously shown in the case of endocarditis [3], our case confirms the value of [^18^F]FDG-PET/CT in diagnosing concealed infections.
